# Is the clinical Queensland High Risk Foot Form valid or reliable for research?

**DOI:** 10.1186/1757-1146-6-S1-O23

**Published:** 2013-05-31

**Authors:** Peter A Lazzarini, Vanessa Ng, Ewan M Kinnear, Maarten C Kamp, Suzanne S Kuys, Cameron Hurst, Lloyd Reed

**Affiliations:** 1 Allied Health Research Collaborative, Metro North Hospital & Health Service, Queensland Health, Brisbane, Queensland, 4032, Australia; 2 Department of Podiatry, Metro North Hospital & Health Service, Queensland Health, Brisbane, Queensland, 4032, Australia; 3 School of Clinical Sciences, Queensland University of Technology, Brisbane, Queensland, 4059, Australia; 4 School of Medicine, The University of Queensland, Brisbane, Queensland, 4072, Australia; 5 Department of Endocrinology, Metro North Hospital & Health Service, Queensland Health, Brisbane, Queensland, 4029, Australia; 6 Centre for Musculoskeletal Research, Griffith Health Institute, Griffith University, Gold Coast, Queensland, 4222, Australia; 7 School of Public Health, Queensland University of Technology, Brisbane, Queensland, 4059, Australia

## Background

High-risk foot complications such as neuropathy, ischaemia, deformity, infections, ulcers and amputations consume considerable health care resources and typically result from chronic diseases. This study aimed to develop and test the validity and reliability of a Queensland High Risk Foot Form (QHRFF) tool.

## Methods

Phase one involved developing a QHRFF using an existing diabetes high-risk foot tool, literature search, expert panel and several state-wide stakeholder groups. Phase two tested the criterion-related validity along with inter- and intra-rater reliability of the final QHRFF. Three cohorts of patients (n = 94) and four clinicians, representing different levels of expertise, were recruited. Validity was determined by calculating sensitivity, specificity and positive predictive values (PPV). Kappa and intra-class correlation (ICC) statistics were used to establish reliability.

## Results

A QHRFF tool containing 46-items across seven domains was developed and endorsed. The majority of QHRFF items achieved moderate-to-perfect validity (PPV = 0.71 – 1) and reliability (Kappa/ICC = 0.41 – 1). Items with weak validity and/or reliability included those identifying health professionals previously attending the patient, other (non-listed) co-morbidity, previous foot ulcer, foot deformity, optimum offloading and optimum footwear.

## Conclusions

The QHRFF had moderate-to-perfect validity and reliability across the majority of items, particularly identifying individual co-morbidities and foot complications. Items with weak validity or reliability need to be re-defined or removed. Overall, the QHRFF appears to be a valid and reliable tool to assess, collect and measure clinical data pertaining to high-risk foot complications for clinical or research purposes.

